# Supplementary material for: Impact of Incorporating Free Calcium and Magnesium on the Heat Stability of a Dairy- and Soy-Protein-Containing Model Emulsion
Source: Polymers (Basel). 2023 Nov 16;15(22):4424. doi: 10.3390/polym15224424 (PMC10675836; doi:10.3390/polym15224424)
Supplement: Supplementary file 1 [file polymers-15-04424-s001.zip › polymers-2635900-supplementary.pdf]

polymers

MDPI

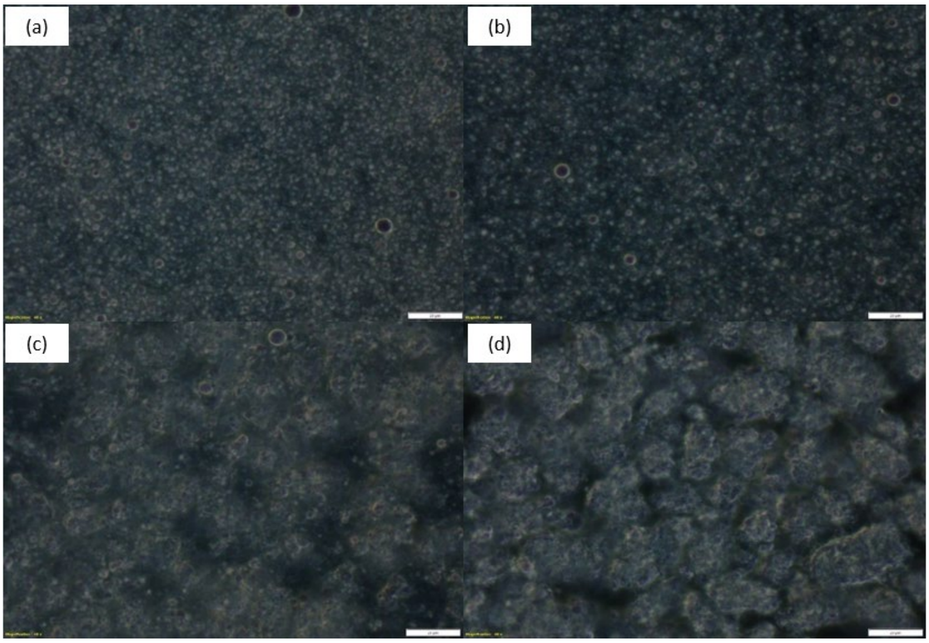

Figure S1. Microscopy of samples with different dose of $CaCl_2$ addition (**a**) 2mM (**b**) 3.5mM (**c**) 5mM (**d**) 6.5mM.

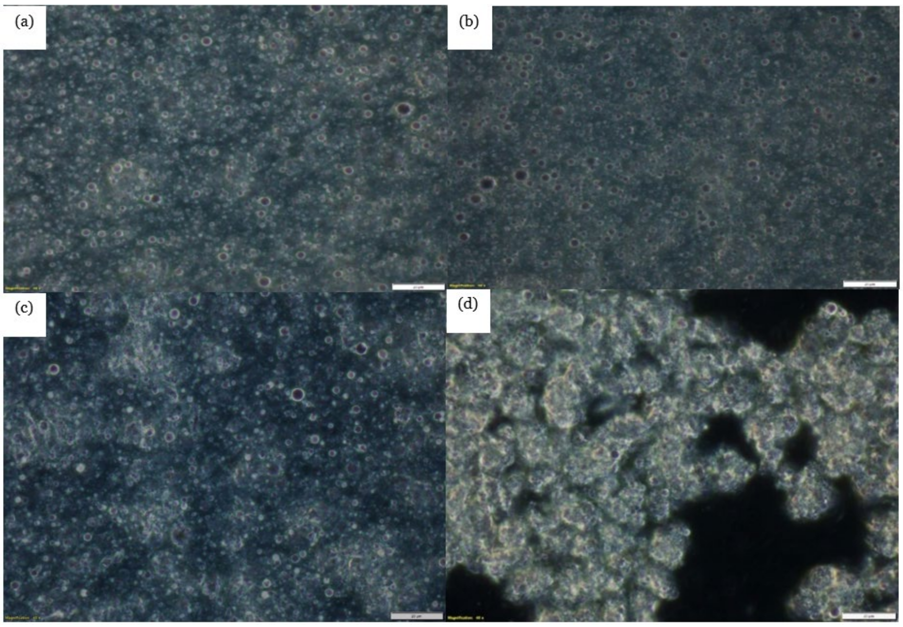

Figure S2. Microscopy of samples with different dose of $MgCl_2$ addition (**a**) 2mM (**b**) 3.5mM (**c**) 5mM (**d**) 6.5mM.
